# WHO guidelines on testing for hepatitis B and C – meeting targets for testing

**DOI:** 10.1186/s12879-017-2765-2

**Published:** 2017-11-01

**Authors:** Margaret E. Hellard, Roger Chou, Philippa Easterbrook

**Affiliations:** 1 Burnet Institute, 85 Commercial Road, Melbourne, Australia; 2 The Alfred Hospital, Melbourne, Australia; 3 Oregon Health & Science University, Portland, OR USA; 4 Global Hepatitis Programme, HIV Department, World Health Organization, Geneva, Switzerland

**Keywords:** Viral hepatitis, Hepatitis C, Hepatitis B, Testing

An estimated 71 million people worldwide are chronically infected with hepatitis C [1] and 257 million with hepatitis B [1–4]. Combined, hepatitis C and hepatitis B are estimated to cause 1.34 million deaths annually, and viral hepatitis is now the 7th leading cause of death globally, ahead of HIV and malaria [2, 3]. The burden of hepatitis B and C disease in 2013 was estimated at 38.7 million disability-adjusted life years (DALYs), an increase of at least 25% since 1990 [3, 4].

In late 2015, world leaders adopted the 2030 Agenda for Sustainable Development, which contains 17 Sustainable Development Goals (SDGs) [4]. The SDGs represent a shift away from the disease–specific goals of the Millennium Development Goals (MDG) era and have adopted an approach where health related goals are embedded across the SDGs. There is also a focus on integration and the aspiration of universal health insurance cover. SDG 3.3 aims to ensure healthy lives and promote well-being at all ages, and highlights the need to combat viral hepatitis [4] (Table 1). In response to the SDGs, the World Health Organization (WHO), working with member states, developed the first-ever *Global health sector strategy on viral hepatitis, 2016–2021*, which was endorsed by the World Health Assembly in May 2016 [5]. WHO’s vision is for “a world where viral hepatitis transmission is halted and everyone living with viral hepatitis has access to safe, affordable and effective prevention, care and treatment services” [5]. The strategy also includes targets for the elimination of hepatitis B and C as public health threats - a 90% reduction in new chronic infections and a 65% reduction in mortality by 2030 from 2015 levels [5]. Achieving these targets will require reaching ambitious service coverage milestones across seven prevention and care interventions, that includes diagnosing 80% of people with chronic viral hepatitis by 2030 and treating eight million people by 2020, and 80% of those eligible for treatment by 2030.

**Table 1:** WHO vison for viral hepatitis and the *Sustainable Development Goal 3.3:* [4, 5]

Vision: “A world where viral hepatitis transmission is halted and everyone living with viral hepatitis has access to safe, affordable and effective prevention, care and treatment services”	
Goal: Eliminate viral hepatitis as a major public health threat by 2030	
SDG 3.3 “End epidemics of AIDS, TB, malaria and neglected tropical diseases and combat hepatitis, water-borne diseases and other communicable diseases”	

These targets are ambitious but achievable. However it is crucial to considerably increase the number of people being tested for viral hepatitis and who are aware of their status if the treatment targets are to be met and the elimination agenda advanced. Currently, it is estimated that only a small proportion of persons with viral hepatitis have been diagnosed - 9% of HBV-infected persons (22 million), and 20% of HCV-infected persons (14 million) globally [1] with the majority diagnosis, and treatments, occuring in higher income settings [6, 7]. In many LMICs, it is estimated that less than 1% of those infected have been diagnosed and treated.

## New WHO testing guidelines

As part of this broader global response to viral hepatitis, and to complement existing care and treatment guidance for HBV [8] and HCV [9], WHO has now developed guidelines on hepatitis B and C testing for low and middle-income countries (LMICs) [10] (Tables 2, 3, 4 and 5). In recognition of the need to substantially increase viral hepatitis testing to meet the 2030 elimination targets (particularly in low and middle-income countries), but also of the substantial cost to health budgets of increased testing, the guidelines take an evidence-based but pragmatic, low-cost approach. Their primary target audience are policy makers responsible for development of national hepatitis testing and treatment programmes in LMICs. A particularly challenging aspect in the guidelines’ development was the limited direct quantity and quality of evidence available to guide the development of recommendations [11] based on the use of the GRADE process. In addition, very few rapid diagnostic serological tests for hepatitis B surface antigen or hepatitis C antibody have undergone formal quality assurance approval process by WHO (pre-qualification) or another recognised stringent national regulatory programme.

**Table 2:** Adaptation (with permission) of Table 1. Summary of recommendations on testing for chronic hepatitis B and C virus infection, from WHO Guidelines on hepatitis B and C testing [10]). Who to test for chronic HBV infection

WHO TO TEST FOR CHRONIC HBV INFECTION	
Testing approach and population	Recommendations^a^
General population testing	1. In settings with a ≥ 2% or ≥5%^b^ HBsAg seroprevalence in the general population, it is recommended that all adults have routine access to and be offered HBsAg serological testing with linkage to prevention, care and treatment services. General population testing approaches should make use of existing community- or health facility-based testing opportunities or programmes such as at antenatal clinics, HIV or TB clinics. *Conditional recommendation, low quality of evidence*
Routine testing in pregnant women	2. In settings with a ≥ 2% or ≥5%%^b^ HBsAg seroprevalence in the general population, it is recommended that HBsAg serological testing be routinely offered to all pregnant women in antenatal clinics^c^, with linkage to prevention, care and treatment services. Couples and partners in antenatal care settings should be offered HBV testing services. *Strong recommendation, low quality of evidence*
Focused testing in most affected populations	3. In all settings (and regardless of whether delivered through facility- or community- based testing), it is recommended that HBsAg serological testing and linkage to care and treatment services be offered to the following individuals: • Adults and adolescents from populations most affected by HBV infection^d^ (i.e. who are either part of a population with high HBV seroprevalence or who have a history of exposure and/or high-risk behaviours for HBV infection); • Adults, adolescents and children with a clinical suspicion of chronic viral hepatitis^e^ (i.e. symptoms, signs, laboratory markers); • Sexual partners, children and other family members, and close household contacts of those with HBV infection^f^; • Health-care workers: in all settings, it is recommended that HBsAg serological testing be offered and hepatitis B vaccination given to all health-care workers who have not been vaccinated previously (*adapted from existing guidance on hepatitis B vaccination* ^*g*^) *Strong recommendation, low quality of evidence*
Blood donors *Adapted from existing 2010 WHO guidance (Screening donated blood for transfusion transmissible infections* ^*h*^ *)*	4. In all settings, screening of blood donors should be mandatory with linkage to care, counselling and treatment for those who test positive.

*Abbreviations: HBsAg* hepatitis B surface antigen, *PWID* people who inject drugs, *MSM* men who have sex with men

^a^The GRADE system (Grading of Recommendations, Assessment, Development and Evaluation) was used to categorize the strength of recommendations as strong or conditional (based on consideration of the quality of evidence, balance of benefits and harms, acceptability, resource use and programmatic feasibility) and the quality of evidence as high, moderate, low or very low

^b^A threshold of ≥2% or ≥5% seroprevalence was based on several published thresholds of intermediate or high seroprevalence. The threshold used will depend on other country considerations and epidemiological context

^c^Many countries have chosen to adopt routine testing in all pregnant women, regardless of seroprevalence in the general population, and particularly where seroprevalence ≥2%. A full vaccination schedule including birth dose should be completed in all infants, in accordance with the WHO position paper on hepatitis B vaccines 2009^g^

^d^Includes those who are either part of a population with higher seroprevalence (e.g. some mobile/migrant populations from high/intermediate endemic countries, and certain indigenous populations) or who have a history of exposure or high-risk behaviours for HBV infection (e.g. PWID, people in prisons and other closed settings, MSM and sex workers, HIV-infected persons, partners, family members and children of HBV-infected persons)

^e^Features that may indicate underlying chronic HBV infection include clinical evidence of existing liver disease, such as cirrhosis or hepatocellular carcinoma (HCC), or where there is unexplained liver disease, including abnormal liver function tests or liver ultrasound

^f^In all settings, it is recommended that HBsAg serological testing with hepatitis B vaccination of those who are HBsAg negative and not previously vaccinated be offered to all children with parents or siblings diagnosed with HBV infection or with clinical suspicion of hepatitis, through community- or facility-based testing

^g^WHO position paper. Hepatitis B vaccines. Weekly Epidemiological Record. 2009;4 (84):405–20

^h^Screening donated blood for transfusion transmissible infections. Geneva: World Health Organization; 2010

**Table 3:** Adaptation (with permission) of Table 1. Summary of recommendations on testing for chronic hepatitis B and C virus infection, from WHO Guidelines on hepatitis B and C testing [10]). Who to test for chronic HCV infection

WHO TO TEST FOR CHRONIC HCV INFECTION	
Testing approach and population	Recommendations^a^
Focused testing in most affected populations	1. In all settings (and regardless of whether delivered through facility- or community- based testing), it is recommended that serological testing for HCV antibody (anti- HCV)^b^ be offered with linkage to prevention, care and treatment services to the following individuals: • Adults and adolescents from populations most affected by HCV infection^c^ (i.e. who are either part of a population with high HCV seroprevalence or who have a history of exposure and/or high-risk behaviours for HCV infection); • Adults, adolescents and children with a clinical suspicion of chronic viral hepatitis^d^(i.e. symptoms, signs, laboratory markers). *Strong recommendation, low quality of evidence* *Note: Periodic re-testing using HCV NAT should be considered for those with ongoing risk of acquisition or reinfection.*
General population testing	2. In settings with a ≥ 2% or ≥5%^e^ HCV antibody seroprevalence in the general population, it is recommended that all adults have access to and be offered HCV serological testing with linkage to prevention, care and treatment services. General population testing approaches should make use of existing community- or facility-based testing opportunities or programmes such as HIV or TB clinics, drug treatment services and antenatal clinics^f^. *Conditional recommendation, low quality of evidence*
Birth cohort testing	3. This approach may be applied to specific identified birth cohorts of older persons at higher risk of infection^g^and morbidity within populations that have an overall lower general prevalence. *Conditional recommendation, low quality of evidence*

*Abbreviations*: *NAT* nucleic acid test, *anti-HCV* HCV antibody, *PWID* people who inject drugs, *MSM* men who have sex with men

^a^The GRADE system (Grading of Recommendations, Assessment, Development and Evaluation) was used to categorize the strength of recommendations as strong or conditional (based on consideration of the quality of evidence, balance of benefits and harms, acceptability, resource use and programmatic feasibility) and the quality of evidence as high, moderate, low or very low

^b^This may include fourth-generation combined antibody/antigen assays

^c^Includes those who are either part of a population with higher seroprevalence (e.g. some mobile/migrant populations from high/intermediate endemic countries, and certain indigenous populations) or who have a history of exposure or high-risk behaviours for HCV infection (e.g. PWID, people in prisons and other closed settings, MSM and sex workers, and HIV-infected persons, children of mothers with chronic HCV infection especially if HIV-coinfected)

^d^Features that may indicate underlying chronic HCV infection include clinical evidence of existing liver disease, such as cirrhosis or hepatocellular carcinoma (HCC), or where there is unexplained liver disease, including abnormal liver function tests or liver ultrasound

^e^A threshold of ≥2% or ≥5% seroprevalence was based on several published thresholds of intermediate and high seroprevalence. The threshold used will depend on other country considerations and epidemiological context

^f^Routine testing of pregnant women for HCV infection is currently not recommended

^g^Because of historical exposure to unscreened or inadequately screened blood products and/or poor injection safety

**Table 4:** Adaptation (with permission) of Table 1. Summary of recommendations on testing for chronic hepatitis B and C virus infection, from WHO Guidelines on hepatitis B and C testing [10]). How to test for chronic HBV infection and monitor treatment response

HOW TO TEST FOR CHRONIC HBV INFECTION AND MONITOR TREATMENT RESPONSE	
Topic	Recommendations^a^
Which serological assays to use	• For the diagnosis of chronic HBV infection in adults, adolescents and children (>12 months of age^b^), a serological assay (in either RDT or laboratory-based immunoassay format^c^) that meets minimum quality, safety and performance standards^d^(*with regard to both analytical and clinical sensitivity and specificity*) is recommended to detect hepatitis B surface antigen (HBsAg). - In settings where existing laboratory testing is already available and accessible, laboratory-based immunoassays are recommended as the preferred assay format. - In settings where there is limited access to laboratory testing and/or in populations where access to rapid testing would facilitate linkage to care and treatment, use of RDTs is recommended to improve access. *Strong recommendation, low/moderate quality of evidence*
Serological testing strategies	• In settings or populations with an HBsAg seroprevalence of ≥0.4%^e^, a single serological assay for detection of HBsAg is recommended, prior to further evaluation for HBV DNA and staging of liver disease. • In settings or populations with a low HBsAg seroprevalence of <0.4%^e^, confirmation of HBsAg positivity on the same immunoassay with a neutralization step or a second different RDT assay for detection of HBsAg may be considered^f^. *Conditional recommendation, low quality of evidence*
Detection of HBV DNA – assessment for treatment *Adapted from existing guidance (WHO HBV 2015 guidelines* ^*g*^ *)*	• Directly following a positive HBsAg serological test, the use of quantitative or qualitative nucleic acid testing (NAT) for detection of HBV DNA is recommended as the preferred strategy and to guide who to treat or not treat. *Strong recommendation, moderate/low quality of evidence*
Monitoring for HBV treatment response and disease progression *Existing guidance (WHO HBV 2015 guidelines* ^*g*^ *)*	• It is recommended that the following be monitored at least annually: - ALT levels (and AST for APRI), HBsAg^h^, HBeAg^i^, and HBV DNA levels (where HBV DNA testing is available) - Non-invasive tests (APRI score or transient elastography) to assess for presence of cirrhosis in those without cirrhosis at baseline; - If on treatment, adherence should be monitored regularly and at each visit. *Strong recommendation, moderate quality of evidence* More frequent monitoring is recommended: • In persons on treatment or following treatment discontinuation: more frequent on- treatment monitoring (at least every 3 months for the first year) is indicated in: persons with more advanced disease (compensated or decompensated cirrhosis^j^); during the first year of treatment to assess treatment response and adherence; where treatment adherence is a concern; in HIV-coinfected persons; and in persons after discontinuation of treatment. *Conditional recommendation, very low quality of evidence* • In persons who do not yet meet the criteria for antiviral therapy: i.e. persons who have intermittently abnormal ALT levels or HBV DNA levels that fluctuate between 2000 IU/mL and 20,000 IU/mL (where HBV DNA testing is available) and in HIV- coinfected persons^h^. *Conditional recommendation, low quality of evidence*

*Abbreviations*: *ALT* alanine aminotransferase, *AST* aspartate aminotransferase, *APRI* aspartate-to-platelet ratio index, *HBeAg* HBV e antigen, *HBsAg* HBV surface antigen, *NAT* nucleic acid test, *RDT* rapid diagnostic test

^a^The GRADE system (Grading of Recommendations, Assessment, Development and Evaluation) was used to categorize the strength of recommendations as strong or conditional (based on consideration of the quality of evidence, balance of benefits and harms, acceptability, resource use and programmatic feasibility) and the quality of evidence as high, moderate, low or very low

^b^ A full vaccination schedule including birth dose should be completed in all infants in accordance with the WHO position paper on Hepatitis B vaccines, 2009. Testing of exposed infants is problematic within the first six months of life as HBsAg and hepatitis B DNA may be inconsistently detectable in infected infants. Exposed infants should be tested for HBsAg between 6 and 12 months of age to screen for evidence of hepatitis B infection. In all age groups, acute HBV infection can be confirmed by the presence of HBsAg and IgM anti-HBc. CHB is diagnosed if there is persistence of HBsAg for six months or more

^c^ Laboratory-based immunoassays include enzyme immunoassay (EIA), chemoluminescence immunoassay (CLIA), and electrochemoluminescence assay (ECL)

^d^ Assays should meet minimum acceptance criteria of either WHO prequalification of in vitro diagnostics (IVDs) or a stringent regulatory review for IVDs. All IVDs should be used in accordance with manufacturers’ instructions for use and where possible at testing sites enrolled in a national or international external quality assessment scheme

^e^ Based on results of predictive modelling of positive predictive values according to different thresholds of seroprevalence in populations to be tested, and assay diagnostic performance

^f^ A repeat HBsAg assay after 6 months is also a common approach used to confirm chronicity of HBV infection

^g^ For further details, *see* Chapter 5: Who to treat and who not to treat. Guidelines for the prevention, care and treatment of persons with chronic hepatitis B infection: World Health Organization; 2015

^h^ In persons on treatment, monitor for HBsAg loss (although this occurs rarely), and for seroreversion to HBsAg positivity after discontinuation of treatment

^i^ Monitoring of HBeAg/anti-HBe mainly applies to those who are initially HBeAg positive. However, those who have already achieved HBeAg seroconversion and are HBeAg negative and anti-HBe positive may serorevert

^j^ Decompensated cirrhosis is defined by the development of portal hypertension (ascites, variceal haemorrhage and hepatic encephalopathy), coagulopathy, or liver insufficiency (jaundice). Other clinical features of advanced liver disease/cirrhosis may include: hepatomegaly, splenomegaly, pruritus, fatigue, arthralgia, palmar erythema and oedema

**Table 5:** Adaptation (with permission) of Table 1. Summary of recommendations on testing for chronic hepatitis B and C virus infection, from WHO Guidelines on hepatitis B and C testing [10]). How to test for chronic HCV infection and monitor treatment response

HOW TO TEST FOR CHRONIC HCV INFECTION AND MONITOR TREATMENT RESPONSE	
Topic	Recommendations^a^
Which serological assays to use	• To test for serological evidence of past or present infection in adults, adolescents and children (>18 months of age^b^), an HCV serological assay (antibody or antibody/antigen) using either RDT or laboratory-based immunoassay formats^c^ that meet minimum safety, quality and performance standards^d^ (*with regard to both analytical and clinical sensitivity and specificity*) is recommended. - In settings where there is limited access to laboratory infrastructure and testing, and/or in populations where access to rapid testing would facilitate linkage to care and treatment, RDTs are recommended. *Strong recommendation, low/moderate quality of evidence*
Serological testing strategies	In adults and children older than 18 months^b^, a single serological assay for initial detection of serological evidence of past or present infection is recommended prior to supplementary nucleic acid testing (NAT) for evidence of viraemic infection. *Conditional recommendation, low quality of evidence*
Detection of viraemic infection	• Directly following a reactive HCV antibody serological test result, the use of quantitative or qualitative NAT for detection of HCV RNA is recommended as the preferred strategy to diagnose viraemic infection. *Strong recommendation, moderate/low quality of evidence* • An assay to detect HCV core (p22) antigen, which has comparable clinical sensitivity to NAT, is an alternative to NAT to diagnose viraemic infection^e^. *Conditional recommendation, moderate quality of evidence*
Assessment of HCV treatment response	• Nucleic acid testing for qualitative or quantitative detection of HCV RNA should be used as test of cure at 12 or 24 weeks (i.e. sustained virological response (SVR12 or SVR24)) after completion of antiviral treatment. *Conditional recommendation, moderate/low quality of evidence*

*Abbreviations*: *DBS* dried blood spot, *IVD* in vitro diagnostics, *NAT* nucleic acid test, *RDT* rapid diagnostic test

^a^The GRADE system (Grading of Recommendations, Assessment, Development and Evaluation) was used to categorize the strength of recommendations as strong or conditional (based on consideration of the quality of evidence, balance of benefits and harms, acceptability, resource use and programmatic feasibility) and the quality of evidence as high, moderate, low or very low

^b^HCV infection can be confirmed in children under 18 months only by virological assays to detect HCV RNA, because transplacental maternal antibodies remain in the child’s bloodstream up until 18 months of age, making test results from serology assays ambiguous

^c^Laboratory-based immunoassays include enzyme immunoassay (EIA), chemoluminescence immunoassay (CLIA), and electrochemoluminescence assay (ECL)

^d^Assays should meet minimum acceptance criteria of either WHO prequalification of IVDs or a stringent regulatory review for IVDs. All IVDs should be used in accordance with manufacturers’ instructions, and where possible at testing sites enrolled in a national or international external quality assessment scheme

^e^A lower level of analytical sensitivity can be considered, if an assay is able to improve access (i.e. an assay that can be used at the point of care or suitable for dried blood spot [DBS] specimens) and/or affordability. An assay with a limit of detection of 3000 IU/mL or lower would be acceptable and would identify 95% of those with viraemic infection, based on available data

The first group of recommendations focuses on who to test for chronic hepatitis B and C. There was a strong recommendation for focussed testing among people most affected by viral hepatitis B or C (defined as those who are either part of a population with higher seroprevalence (e.g. some mobile/migrant populations from high/intermediate endemic countries, some indigenous populations) or who have a history of exposures or high-risk behaviours for HCV infection (e.g. PWID, people in prisons and other closed settings, MSM and sex workers, partners, family members and children of HBV infected persons) as well as adults and children with a clinical suspicion of chronic viral hepatitis. The guidelines made a conditional recommendation that in settings with intermediate (≥2%) or high (≥5%) prevalence, all adults should have routine access to testing, with linkage to care and prevention services (Tables 2, 3, 4 and 5). The threshold used will depend on other country considerations and epidemiological context. In settings in which there are defined birth cohorts of older patients at higher risk for hepatitis C infection, the guidelines also recommend consideration of routine testing in birth cohorts. The strength of these recommendations was conditional, reflecting the more limited and lower-quality supporting evidence. Importantly, the guidelines recognise that testing should make use of existing community or health facility-based testing opportunities or programmes such as at antenatal clinics, HIV or TB clinics [10] (Tables 2, 3, 4 and 5). The guidelines provide illustrative examples of different testing service delivery models in different populations and settings.

The second group of recommendations addressed “how to test?” with respect to choice of assays (i.e enzyme immunoassays (EIA) that are generally performed in a laboratory setting compared to rapid diagnostic tests (RDTs) that can be undertaken within the community) and sequence of tests (ie. testing strategies). Although RDTs generally are slightly less accurate than EIAs, they may enhance access to testing in settings with poor access to laboratory testing and facilitate receipt of results and linkage to care and treatment. The trade-off between the small difference in test accuracy and the key goal to promote access to testing, means that on balance EIA was recommended as the preferred assay in settings where laboratory testing is available, especially for HBsAg where there is a wide variation in diagnostic performance of available RDT assays, and RDT recommended in settings with poor access to laboratory testing and/or in populations where access to rapid testing would facilitate linkage to care and treatment. The recommendation was graded “strong”, based on low/moderate quality of evidence [10] (Tables 2, 3, 4 and 5).

The need for a one or two-assay serological testing strategy was also addressed. Again, whilst a second confirmatory test would improve diagnostic accuracy, particularly in lower prevalence settings, this would incur additional complexity and costs. This led to the pragmatic recommendation that a single initial RDT or EIA was sufficient prior to a supplementary nucleic acid test (NAT) test for current viraemia. In low-prevalence settings (≤0.4%), confirmation of hepatitis B surface antigen (HBsAg) with a neutralisation step or a second and different RDT HBsAg assay should be considered due to the considerably improved positive predictive value (and hence reduced false positive rate) they confer. The strength of this recommendation was “conditional”, based on low quality of evidence [10] (Tables 2, 3, 4 and 5).

The use of a qualitative or quantitative nucleic acid test (NAT) to detect viraemia and inform assessment of an individual’s need for hepatitis B or C treatment was recommended (strong recommendation, moderate/low quality of evidence). With highly effective curative short course direct acting antiviral (DAA) treatment now available for hepatitis C infection, with need only to confirm presence or viraemia and cure, a qualitative test may be sufficient depending on the limit of detection. For hepatitis C, a core antigen test with comparable clinical sensitivity (and potentially a simpler test for some laboratories) was recommended as a potential alternative to NAT for diagnosis of viraemic infection (conditional recommendation, moderate quality of evidence). When assessing treatment response and test of cure for hepatitis C, a NAT test was recommended rather than an antigen test for which there is currently insufficient data [10] (Tables 2, 3, 4 and 5). Monitoring for hepatitis B was addressed in the previously developed WHO Hepatitis B Guidelines [8] and includes annual HBsAg and HBV DNA measurement.

The use of dried blood spots (DBS) specimens for HBsAg and anti-HCV antibody serology testing and HBV and HCV NAT was examined. Again, trade-offs were considered, specifically whether whether DBS testing would sufficiently increase the number of tests being performed to an extent that would offset the reduced sensitivity and specificity. DBS testing was recommended where there are no facilities or expertise to take venous whole blood specimens; for persons with poor venous access (e.g. people who inject drugs); or where RDTs were not available or their use was not feasible. It was recognised that a key limitation to the expanded use of DBS was that currently there are no manufacturers’ protocols for use of DBS samples with their commercial assays, or regulatory approval for their use with DBS samples. As a consequence the current use of DBS specimens would be considered “off-label”. The recommendation (conditional, with low/moderate quality evidence) highlights the need to strengthen our understanding of the benefits and limitations of using DBS [10] (Tables 2, 3, 4 and 5).

For testing to improve patients’ outcomes it is necessary that patients testing positive are linked to care and receive appropriate treatment. Recommended strategies to improve linkage to care following a positive serological test for hepatitis B or C include the role of peers and lay health workers, clinician reminders to prompt testing, integration of testing into single one-stop-shop facilities such as mental health or drug services, and on-site RDT testing with same-day results. However, specific evidence that following hepatitis testing, a support service or service structure improves linkage to care and treatment are limited so the recommendation was graded conditional with low/moderate quality evidence [10] (Tables 2, 3, 4 and 5).

These inaugural viral hepatitis testing guidelines represent an important first step on the road map for increasing access to hepatitis B and C testing and to support the elimination goal. It also provides general guidance to countries on how to implement the recommendations and strategically select testing approaches and services and organise their laboratory services.

A challenge has been the quantity and quality of data to inform the testing recommendations, and these guidelines highlight the evidence and research gaps and agenda for the future. This should help governments in their decision-making on how best to implement testing programs. Hopefully it will also encourage manufacturers to register and qualify their RDTs and DBS tests, making it easier for services to utilise them. Demonstration/implementation science projects are needed to further guide implementation at country and regional level according to country epidemic profile and health services context.

Elimination of hepatitis B and hepatitis C as public health threats by 2030 is a laudable and feasible goal. The WHO testing guidelines will inform elimination strategies at individual health services and at country and regional levels. In addition, they will provide impetus for the development of the low-cost, high-quality tests that are vital for meeting elimination targets.
